# Multifunctional Adhesives on the Eggs of the Leaf Insect *Phyllium philippinicum* (Phasmatodea: Phylliidae): Solvent Influence and Biomimetic Implications

**DOI:** 10.3390/biomimetics5040066

**Published:** 2020-11-27

**Authors:** Thies H. Büscher, Raunak Lohar, Marie-Christin Kaul, Stanislav N. Gorb

**Affiliations:** Department of Functional Morphology and Biomechanics, Institute of Zoology, Kiel University, Am Botanischen Garten 9, 24118 Kiel, Germany; ralo@tf.uni-kiel.de (R.L.); stu119303@mail.uni-kiel.de (M.-C.K.); sgorb@zoologie.uni-kiel.de (S.N.G.)

**Keywords:** insect glue, attachment biomechanics, walking leaves, fiber reinforcement

## Abstract

Leaf insects (Phylliidae) are well-camouflaged terrestrial herbivores. They imitate leaves of plants almost perfectly and even their eggs resemble seeds—visually and regarding to dispersal mechanisms. The eggs of the leaf insect *Phyllium philippinicum* utilize an adhesive system with a combination of glue, which can be reversibly activated through water contact and a water-responding framework of reinforcing fibers that facilitates their adjustment to substrate asperities and real contact area enhancement. So far, the chemical composition of this glue remains unknown. To evaluate functional aspects of the glue–solvent interaction, we tested the effects of a broad array of chemical solvents on the glue activation and measured corresponding adhesive forces. Based on these experiments, our results let us assume a proteinaceous nature of the glue with different functional chemical subunits, which enable bonding of the glue to both the surface of the egg and the unpredictable substrate. Some chemicals inhibited adhesion, but the deactivation was always reversible by water-contact and in some cases yielded even higher adhesive forces. The combination of glue and fibers also enables retaining the adhesive on the egg, even if detached from the egg’s surface. The gained insights into this versatile bioadhesive system could hereafter inspire further biomimetic adhesives.

## 1. Introduction

Phasmids—also called stick and leaf insects—render proof for a highly connected co-evolution between these herbivore insects and plants. Different aspects of their morphology and physiology, e.g., cryptic masquerade [1,2], oviposition [2,3,4,5,6], as well as their elaborate tarsal attachment systems [7,8,9,10], substantiate this assertion. Very well-known examples for this aspect of phasmatodean evolution are the Walking Leaves (Phyllidae). These insects perfectly imitate leaves of plants and blend with their environment [11,12,13]. This is substantially made possible by their abdominal lateral extensions and their green coloration (see, e.g., in [14,15,16,17,18]). The first indications for the evolution of plant imitation in Phasmatodea in general date back to the early Cretaceous period [19]. During that period, the floral communities primarily consisted of gymnosperms that were imitated by the stick insects even back then to hide from predators [19]. In the subsequent radiation of angiosperms, both plants and stick insects evolved foliaceous forms, a result of the imitation of the plants by stick insects and particularly leaf insects. 

In addition to the specific body appearance, phasmids established several functional principles similar to plants—particularly to their seeds [2,3,20,21,22]. Some eggs of stick insects deploy similar mechanisms for distribution like plant seeds. This includes endozoochory, particularly by birds, which ingested eggs and disperse them [23,24,25,26], and dispersal via the ocean [27,28,29,30]. Many eggs of stick insects furthermore bear a proteinaceous cap on their operculum (the capitulum). This structure is functionally analogous to the elaiosomes of plant seeds—an attractant for ants which promotes dispersal via myrmecochory [2,3,31,32,33,34]. Another common feature for seed dispersal is the presence of surface glues. Many plants deploy polysaccharides (e.g., cellulose, pectins, or hemicelluloses) in order to either attach their seeds to a suitable place for germination [35,36,37], or to enable zoochoric dispersal of the seeds [23,24,25,26,38,39,40]. Insects in general often employ glues to attach their eggs [22,41,42,43,44]. Attaching the eggs onto their food source is a common principle in phytophagous insects [41,42,43,45], or ectoparasites [46,47]. Many stick insect species either glue their eggs onto the leaves or the bark of plants or pierce them into the leaves [2,3,4,5,6,8,10]. Some insects specifically exploit the complexity of the structures present on the surfaces they deposit their eggs on (e.g., trichomes and wax crystals of leaves [41,43]). Just a few studies report insects with adhesives on their eggs that response to liquids, for example dragonflies that deposit their eggs into the water [48,49,50,51,52,53,54,55,56,57,58]. An adhesive that swells after deposition in water coats these eggs [58]. Besides Odonata, similar egg coatings, which are also activated by water contact, are present in Ephemeroptera [59]. All these species are merolimnic, consequently, their offspring is dependent on the attachment underwater. Adhesion of these eggs underwater relies on water-responding exochorionic structures of the eggs [58,59].

**Figure 1:**
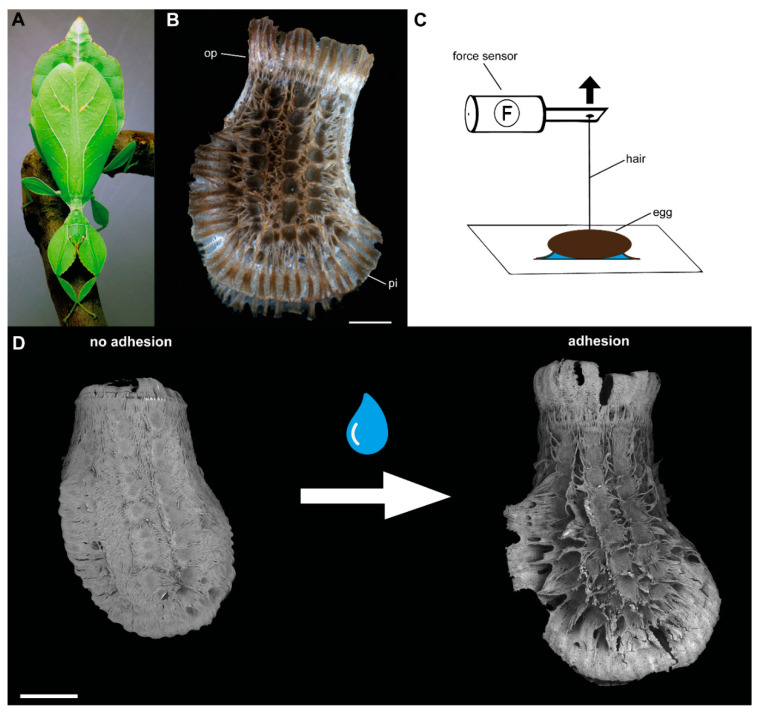
Object of the study and experimental set-up: (**A**) *Phyllium philippinicum* female, adult (modified from the work in [22], provided by Daniel Dittmar). (**B**) Lateral view of an attached egg. (**C**) Set-up of the experiment. The eggs were pulled off a glass plate in the perpendicular direction and the force was measured with a force sensor attached to the egg (modified from the work in [22]). (**D**) Transition from freshly laid eggs, to functional adhesive eggs with expanded pinnae after contact with water. op, operculum; pi, pinna. Scale bars: 1 mm (**B**,**D**).

A similar mechanism has been reported for the eggs of the Philippine leaf insect (*Phyllium philippinicum* Hennemann, Conle, Gottardo and Bresseel, 2009, Figure 1A), which is in contrast terrestrial, but dependent on its foodplants. The eggs (Figure 1B) of this insect are covered with an adhesive secretion, which enables an elaborate multifunctional attachment system [22]. The glue itself responds to water and first liquefies, subsequently spreads into surface corrugations, thus maximizes the actual contact area with the substrate and finally solidifies. This process generates strong adhesion (up to 4800 times the weight of the egg itself; [22]). Furthermore, repetitive water exposure reactivates liquefaction and consequently the mobilization of the glue deactivating the adhesive ability of the glue. The re-liquefied glue can now make contact again and re-solidify. In addition to this reversible glue, the exochorionic morphology supports incubation site optimization and surface attachment by the usage of hierarchically split surface structures, called pinnae. Mentioned pinnate egg structures respond to water similarly as the glue: they expand and spread, the glue can then adapt to different degrees of structure roughness and provide a structurally enhanced adhesive egg surface. The result is a more efficient glue reservoir on the egg surface for subsequent attachment events. Although this mechanism is so far just experimentally tested for the eggs of *P. philippinicum* and, to our knowledge, not mentioned for any other phylliid species, it is likely that such a mechanism is present in other species as well, as similar expendable structures are found on the surface of the eggs of other walking leaf species [14,15,16,17,18]. 

Most insect glues—either on their eggs or for other purposes—are largely proteinaceous [60,61,62,63,64,65,66,67,68,69]. The majority of these adhesives, which were experimentally tested so far, generate strong adhesion to different natural substrates [22,41,42,43,44]. The asparagus beetle (*Crioceris asparagi* (Linnaeus, 1758), Coleoptera: Chrysomelidae) attaches its eggs onto *Asparagus officinalis* L. (Asparagaceae). This plant is coated by wax crystals and therefore has a microstructured superhydrophobic surface. The eggs of this beetle still adhere very well on this challenging surface, due to the composition of the glue [43]. The composition of the egg glue of *P. philippinicum*, however, remains unknown. Previous results suggest a proteinaceous nature (positive methylene blue staining [22]), presumably with at least two functional groups—one polar towards the substrate and the other nonpolar towards the egg surface, as water can activate the adhesion, but does not remove the glue from the egg. Glycoproteins, the molecules involved in such tasks in many other insects [60,70], can fulfill this function. Glycan is soluble in polar solvents and presumably faces to the exterior of the egg towards the substrate [60,68,71], while the hydrophobic protein component adheres to the hydrophobic surface of the egg [72,73]. Only very few of the examined egg glues in the literature were water-soluble (see, e.g., in [60]), others only using strong solvents or acids. 

Although the adhesives of many insects either respond to contact to liquids (see, e.g., in [59]) or are particularly adapted to perform on specific surface chemical properties (see, e.g., in [43]), usually the only comments of liquid glue interactions found in the literature are approaches to dissolve the adhesives for chemical analyses. Experimental approaches, elucidating the attachment performance so far, did not investigate influences of solvents on the glue. Most insect glues are not depending on water activation, especially for terrestrial species, and are water-insoluble, therefore this aspect of adhesive systems does not play a role for most insects. The glue of *P. philippinicum*, in contrast, necessitates water contact to facilitate the function of the glue [22]. However, so far the only solvent type used in experiments is a polar, protic solvent (water). Other chemical solvents, such as nonpolar (e.g., alkanes), aprotic (e.g., ketones) solvents, or solvents with different solvent properties (e.g., chloroform vs. acetone) or different pH can potentially affect the function of the adhesive system [74]. The different chemicals can have an impact on (1) the reaction of the pinnae, (2) the bonding formation of the glue with the substrate, (3) the anchorage on the egg, (4) the denaturation of the glue, and many more effects. Depending on the response to different solvents, conclusions on the chemical nature of the egg glue of *P. philippinicum* and its physical properties can be drawn. To further characterize the composition and functionality of this bioadhesive, different experiments on solvent interaction and attachment force were conducted. 

We tested the response of both glue and eggshell to solvents with different chemical properties (polar, nonpolar, protic, aprotic, etc.) and measured the resulting adhesive force mechanically. Although many adhesive mechanisms of insect eggs require hydration to obtain an adhesive effect [48,49,50,51,52,53,54,55,56,57,58,59], most studies deal solely with the interaction between water and the adhesive. The influence of chemical aspects of the environment is, at least to our knowledge, so far not examined for glue-based adhesives of insect eggs. The response to substances with different chemical properties can not only reveal functional adaptations to the exposition to different liquids, the eggs face in their environments, but, furthermore, yield information about the chemical properties of the glue itself.

Besides using various chemicals, we explored the nature of solvent–glue interaction with

- Repetitive measurements of a sequence of attachment and detachment cycles;
- Comparative series of different pH ranges and different standard solvents (ethanol, acetone, chloroform, toluol, hexane, etc.);
- Repetitive measurement cycles of cross-observations between the solvent and water.

To evaluate the effects of the solvents on the morphology of the exochorionic extensions of the eggs and the glue coverage, we examined the adhesive system of the eggs using scanning electron microscopy (SEM).

We specifically aimed to answer the following questions.

1. Does the solvent influence pinnae reaction and glue liquefaction and consequently attachment performance?
2. Do the attachment forces of eggs differ among solvent treatments?
3. Do different solvents affect the interaction of the glue in its subsequent contact with water?

The elucidation of this multifunctional, multicomposite adhesive system hopefully inspires subsequent studies on the biomolecular composition of the glue and features a potential for bioinspired adhesive applications. The combination of structural features responding to external stimuli (in this case water) and affecting the glue behavior might be interesting for future biomimetic applications to responsive multifunctional glues [60,75,76,77].

## 2. Materials and Methods

### 2.1. Specimens

Fresh eggs of *Phyllium philippinicum* Hennemann, Conle, Gottardo and Bresseel, 2009 (Figure 1A) were used for this study. The eggs were collected from female insects from the culture of Dr. Kirsten Weibert (Jena, Germany). The insects were fed with leaves of bramble (*Rubus* sp.) ad libitum and were exposed to a natural day/night cycle. All eggs were weighed soon after oviposition with the analytical balance AG204 Delta Range microbalance (Mettler Toledo, Greifensee, Switzerland; d = 0.1 mg).

### 2.2. Morphology

Overview images of the eggs were obtained with a Leica M205 binocular microscope (Leica Microsystems Ltd., Wetzlar, Germany) and a Leica DFC420 microscope camera (Leica Microsystems Ltd., Wetzlar, Germany). Multifocus stacked images were post-processed using the software Leica Application Suite (LAS) version 3.8.0 (Leica Microsystems Ltd., Wetzlar, Germany). 

Further overview images were obtained using the scanning electron microscope (SEM) Hitachi TM300 (Hitachi High-technologies Corp., Tokyo, Japan). Both fresh and previously experimentally tested eggs were airdried and sputter-coated with a layer of gold-palladium with 10 nm thickness. Afterward, the eggs were mounted on aluminium stubs and observed at 15 kV acceleration voltage. Micrographs were taken in different focus settings and postprocessed using Photoshop CS6 (Adobe Systems Inc., San Jose, CA, USA). Detailed images of the eggs were obtained using the SEM Hitachi S4800 (Hitachi High-technologies Corp., Tokyo, Japan) at higher magnifications and an acceleration voltage of 5 kV. 

### 2.3. Detachment Force Measurements

The force required to detach individual eggs from the test surface was measured in two different experimental set-ups. For all measurements, single eggs were mounted on clean microscope glass slides (Carl Roth GmbH & Co. KG, Karlsruhe, Germany), as described in [22]. A horsehair was fixed with molten bee wax to the lateral sides of individual eggs (Figure 1C) and connected to a force transducer (100 g capacity; FORT100, World Precision Instruments Inc., Sarasota, FL, USA). The signal of the force transducer was processed with a BIOPAC Model MP100 and a BIOPAC TCI-102 system (BIOPAC Systems, Inc., Goleta, CA, USA). Force–time curves of the detachment process were recorded with the software Acqknowledge 3.7.0 (BIOPAC Systems Inc., Goleta, CA, USA). The detachment force was measured by pulling the test surface away from the sensor at an angle of 90° and with a speed of ~1–2 cm/s using a laboratory lifting platform. The highest peak of the obtained graph was considered the maximum detachment force (see in [22,78,79]). All experiments were performed at 20–22 °C temperature and 40–60% relative humidity. 

#### 2.3.1. Sequential Detachment 

To measure the influence of the solvent on the adhesive system of eggs, freshly laid eggs were placed in different solutions (Table 1) overnight on a shaker. In addition to 100% solutions of different solvents, a 25% solution of glutaraldehyde was used. The chemicals were selected according to their chemical properties, to cover protic, aprotic, polar and apolar solvents, different pH ranges, as well as tanning fixatives, which all can potentially affect the properties of the egg glue. To prepare chemical solutions with desired pH values, acetic acid, and potassium hydroxide (KOH) solution were mixed to produce solutions with gradually increasing pH (2.9, 5.0, 7.0, 10.9, 13.9). The eggs were prepared by removing them from the solution and placing them in a single droplet (~100 μL) of the respective solvent on fresh microscope slides. Subsequently, they were allowed to dry for ~24 h and finally attached to the sensor. The number of eggs adhering to the glass plate was counted, contrasted to the eggs detached by inverting the glass plate and the maximum detachment force was measured for individual eggs as described above. After carrying out the force measurement, the eggs were again treated with the solvent and subsequently mounted on another microscope slide. This procedure was repeated with the same egg for 10 cycles.

#### 2.3.2. Solvent Cross Treatment

Additionally, solvents, which caused no adhesion effect during our preliminary test, were treated the same way as in sequential detachment experiments, but instead of repeating several cycles of treatment with the same solvent, the eggs were deposited in a single droplet of bidistilled water on a new microscope slide (without storage overnight). Subsequently, the egg treatments were alternated between water and the specific solvent, to uncover replicability of glue inhibition and reversibility of adhesion.

### 2.4. Statistical Analysis

SigmaPlot 12.0 (Systat Software Inc., San José, CA, USA) was used for statistical tests. Normal distribution was tested using Shapiro–Wilk’s test and homoscedasticity was tested using Levene’s test. As the data were nonparametric and showed non-homoscedasticity, the maximum attachment forces between the solvents, between solvent and water cross treatments, as well as the initial attachment forces in the sequential force measurements, were compared using Kruskal–Wallis One Way Analyses of Variance (ANOVAs) on Ranks followed by Dunn’s post hoc tests. The different cycles of sequential detachment force tests within the same solvent were compared using Friedman’s Repeated Measures ANOVA on Ranks, followed by Tukey’s post hoc test. The detachment forces of eggs treated with glutaraldehyde and subsequently with water exposure were compared using Wilcoxon Signed Rank Test.

## 3. Results

### 3.1. Sequential Attachment 

Different solvents revealed essential differences in their effects on various aspects of egg attachment. Table 2 provides an overview of the general effects among the solvents. The choice of the solvent had strong effects on the response of the pinnae and the glue (see Section 3.1.2), as well as on the initial detachment force (Table 2). Chloroform, hexane, propan-2-ol, and toluol revealed no adhesion in the initial detachment test and were therefore not sequentially tested with the same solvents, but alternatingly tested with the water treatment (see Section 3.2).

#### 3.1.1. Detachment Force Progression

**Figure 2:**
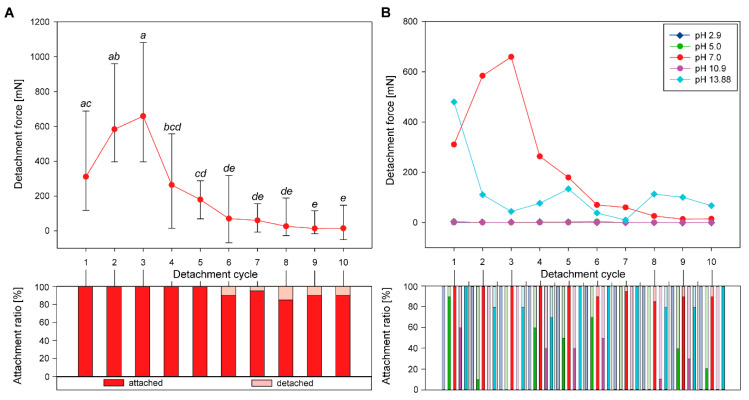
Sequential detachment force measurements of *P. philippinicum* eggs: (**A**) Line plot (above) and the corresponding count of attached and detached eggs (attachment ratio, below) for sequential measurements of eggs exposed to pH 7.0. Error bars indicate s.d., dots represent the median. Groups with the same letter are statistically not different (Friedman repeated measurement ANOVA on ranks, Tukey’s post hoc test, *p* < 0.05); (**B**) Line plots (above) and the corresponding attachment ratio (below) for sequential measurements of eggs exposed to different pH values (line plots for pH 2.9 and 5.0 largely overlap with pH 10.9, see Figure 4A for details).

**Figure 3:**
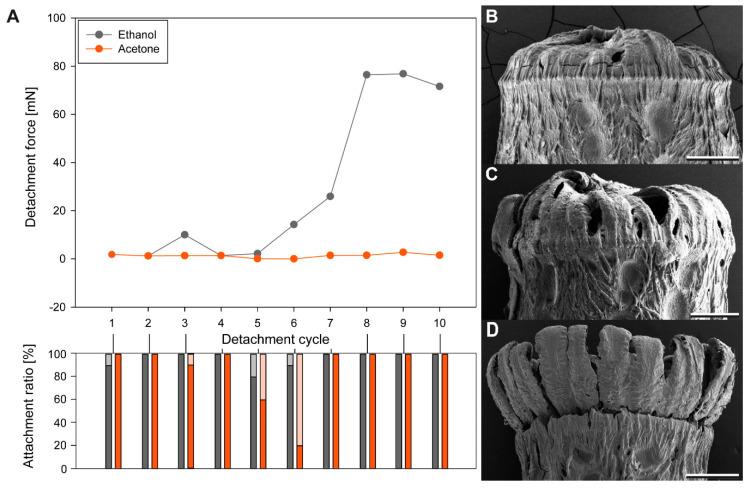
Solvent influence on sequential detachment of freshly laid (dry) eggs: (**A**) Line plots (above) and the corresponding attachment ratio (below) for sequential measurements of eggs exposed to ethanol and acetone; (**B**–**D**) Scanning electron micrographs of the response of the pinnae to solvent exposition: (**B**) Inhibited expansion; (**C**) Moderate expansion; (**D**) Expansion; Scale bars: 500 µm.

The eggs treated with pH 7.0 solution adhered well during the first attachment cycles. Nearly all eggs attached in any repeated attachment cycle (Figure 2A). During the first three cycles, the detachment force of these eggs increased from 310.48 ± 278.33 mN (median ± s.d.) in the initial cycle to 659.13 ± 334.75 mN in the third cycle. In subsequent detachment cycles, the detachment force significantly decreased (Friedman Repeated Measures ANOVA on Ranks, *χ^2^* = 130.13, d.f. = 9, *p* ≤ 0.001; Tukey’s post hoc test, *p* < 0.05). During the last cycles (cycle 6–10), the detachment force was the lowest with median detachment force ranging from 13.56 ± 64.30 mN (cycle 9) to 69.71 ± 188.63 mN (cycle 6) and did not differ statistically significant from each other (Friedman Repeated Measures ANOVA on Ranks, *χ^2^* = 130.13, d.f. = 9, *p* ≤ 0.001; Tukey’s post hoc test, *p* > 0.05). During these cycles, the majority of the eggs securely attached; however, a small amount (5–15%) did not attach in the first place. The progressions of the detachment forces revealed different curves for the other tested pH regimes (Figure 2B). In contrast to the detachment force sequence of pH 7.0 treated eggs, the detachment forces of eggs treated with pH 13.9 were highest in the first cycle and then decreased and remained similarly low (Figure 2B). The attachment ratio of the eggs was similarly high for pH 13.9. All other pH regimes revealed rather low attachment ratios for all measurements, however, except for the lowest pH regime (pH 2.9), all treatment groups showed considerable numbers of attached eggs. Although several eggs attached to the substrate, the detachment forces for pH 5.0 and pH 10.9 were low in all cycles. Likewise, most eggs treated with acetone and ethanol, respectively, attached in all cycles—almost all cycles reached 100% attachment ratio for both solvents (Figure 3A). However, in contrast to the eggs treated with ethanol, the eggs treated with acetone only revealed low detachment forces during all cycles (median detachment force ranging from 0.01 mN to 2.72 mN). The median detachment forces, exhibited by ethanol treated eggs, were low in the first cycles (1.8 mN to 10.0 mN, cycles 1–5), but increased to 25.9 mN to 76.85 mN in cycles 7–10 (Figure 3A).

**Figure 4:**
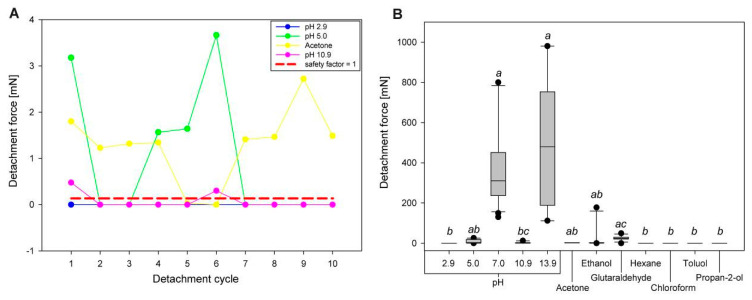
Solvent influence on attachment: (**A**) Line plots for sequential measurements of eggs exposed to different solvents, which revealed low detachment forces. The dashed red line represents a safety factor of 1, or a weight force of 0.136 mN (median weight of all eggs measured); (**B**) Boxplots of the initial detachment force (during the first cycle) of the eggs treated with all solvents used in sequential attachment experiments. The horizontal line represents the median, the upper and lower borders of the boxes represent 25 and 75 percentiles and the whiskers represent 10 and 90 percentiles. Groups with the same lowercase letter are statistically not different (Kruskal–Wallis ANOVA on ranks, Dunn’s post hoc test, *p* < 0.05).

The sequential attachment forces were low in all treatments with acetone and the pH regimes 2.9, 5.0, and 10.9. Figure 4A shows the sequential detachment cycles for these solvents, to compare their detachment force progressions and to contrast these with the adhesive force required for one egg to attach itself to a substrate. The safety factor (*SF*) reflects the adhesive force produced per body weight ($SF=\frac{F\left(detachment\right)}{F_{G}})$. Consequently, a *SF* of 1 corresponds to the force required to attach one egg. As the median weight of the eggs was 13.9 mg (*n* = 144), an *SF* of 1 means 0.136 mN detachment force is the required force to attach one egg (Figure 4A). Most detachment forces, even the lower forces in the sequential tests of different solvents, were sufficient to attach one egg. Just pH 2.9 exhibited only forces not sufficient for single egg attachment. All other sequential test groups produced enough adhesion, at least for a few detachment cycles. The samples treated with pH 10.9 revealed sufficient adhesion (*SF* ≥ 1) during the cycles 1 and 6, those with pH 5 in the cycles 1, 4, and 6 and acetone treated samples adhered in all cycles besides the cycles 5 and 6 (Figure 4A). Although the detachment force measured for these eggs is much smaller than for other treatments, the attachment was sufficient to adhere several times their own weight. Still, the adhesion of the eggs treated with pH 7.0 and 13.9 revealed multiple times higher *SF* already during the first attachment cycles, with initial attachment forces (during the first cycle) of median *SF* of ~2282 (pH 7.0) and 3527 (pH 13.9). 

#### 3.1.2. Comparison of Initial Detachment Forces

The initial detachment forces provide information on how strong the attachment of the eggs was, based on just the first treatment of the particular solvent (Figure 4B). Two groups revealed high detachment forces during the initial attachment cycles (pH 7.0; 310.48 ± 278.33 mN, *n* = 20 and pH 13.9; and 479.78 ± 310.43 mN, *n* = 10). These were statistically significantly higher than most other solvent treatments (Kruskal-Wallis ANOVA on ranks, *H* = 117.07, d.f. = 11, *p* ≤ 0.001; Dunn’s post hoc test, *p* < 0.05). The initial detachment forces for the solvents with pH 2.9, pH 10.9, hexane, chloroform, propan-2-ol, and toluol were significantly lower than those with pH 7.0 and 13.9 (Dunn’s post hoc test, *p* < 0.05). All other treatment solvents resulted in median detachment forces between these two extremes and were not significantly different from both cases (Dunn’s post hoc test, *p* > 0.05). 

#### 3.1.3. Egg Responses to Solvents 

Different solvents had different effects on the two major components of the adhesive system of the eggs, the glue and the pinnae, as revealed by scanning electron microscope analysis of the treated samples. Table 2 summarizes the main effects. Depending on the solvent, the pinnae of the eggs were either regularly expanded, like in contact with water (Figure 3B), expanded to a reduced extent (Figure 3C), or the expansion was completely inhibited (Figure 3D). All pH regimes, except for pH 10.9, resulted in a complete expansion of the egg pinnae, acetone did as well. Only ethanol exhibited a reduced unfolding of the pinnae. All remaining solvents inhibited the expansion of the pinnae. Subsequent treatment with water caused the pinnae to expand again. 

**Figure 5:**
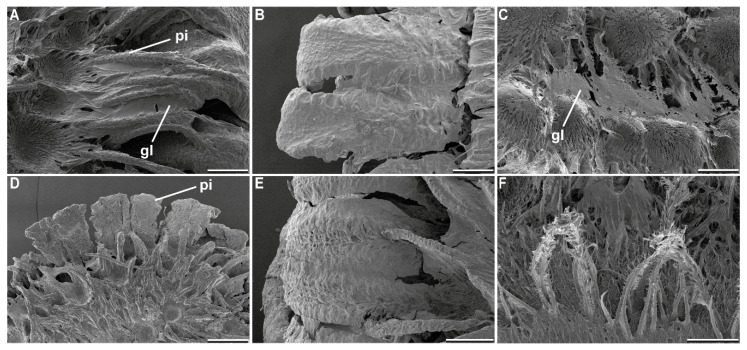
Scanning electron micrographs of the glue morphology after exposition to different solvents: (**A**) Propan-2-ol treated, no response of the glue; (**B**,**D**,**E**) Treatment with high and low pH solutions, denaturation: (**B**) pH 5.0, (**D**) pH 10.9, (**E**) pH 13.9; (**C**) Chloroform, glue solved from the egg surface, but large amounts kept between the pinnae; (**F**) Acetone, glue solved from the most of the egg surface, but residuals kept on the pinnae; pi, pinna; gl, glue; Scale bars: 500 µm (**D**), 200 µm (**A**–**C**,**E**), 100 µm (**F**).

The glue coating of the eggs revealed a different pattern than the pinnae reaction to the solvent (Table 2). The solvents pH 7.0 and ethanol revealed the same behavior as already described for water [22]. These two solvents caused liquefaction of the glue, which then spread over the pinnae and resolidified. All other pH regimes beside the neutral value (pH 7.0) caused a change of the structure of the glue. Instead of becoming fluid, the glue seemingly denatured (Figure 5B,D,E). The glue remained in the original place on the egg, but apparently increased in volume (Figure 5B). Furthermore, the surface structure of the adhesive became less smooth than in the other treatments, but looked crumbly (Figure 5E). Acetone, chloroform, and toluol, however, dissolved the glue from the surface of the egg (Figure 5C,F). The degree of dissolution varied in the three solvents: chloroform treatment caused just a minor relocation of the solvent (Figure 5C). Here, some amount of the glue was detached from the egg surface and gathered on the exterior pinnae, slightly increasing the volume of the glue towards the outer face of the egg. Acetone caused a larger detachment of the glue; however, some amounts of glue remained on the surface of the pinnae due to the hierarchical organization of these structures (Figure 5F). Toluol revealed an intermediate degree of dissolution. All other solvents did not reveal changes of the glue. The glue did seemingly not interact with the solvent at all (Figure 5A).

### 3.2. Sequential Solvent Cross-Treatment 

#### 3.2.1. Progression of Sequential Detachment Forces

**Figure 6:**
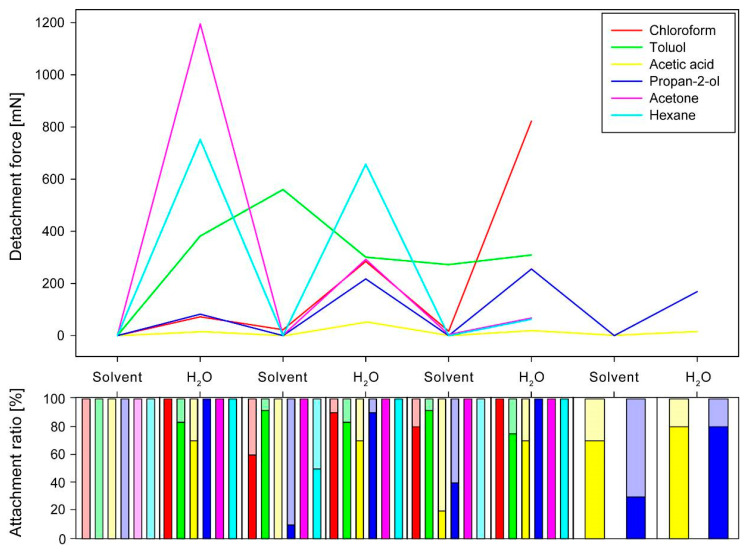
Sequential detachment force measurements of *P. philippinicum* eggs in sequential cross treatment experiments. Line plot (above) and the corresponding count of attached and detached eggs (attachment ratio, below) for sequential measurements of eggs alternatingly exposed to different solvents and water.

The choice of the solvent had a strong influence on the detachment forces during the measurements after exposition to the solvent. However, there was another influence of the solvent to the detachment force of the eggs subsequentially exposed to water. All solvents, tested in the sequential solvent cross treatment experiments, inhibited substantial adhesion of the eggs during the first cycle (Figure 6). This effect was reversible for all the solvents tested. There was no solvent irreversibly inhibiting the adhesion, as all eggs exposed to water after the solvent treatment revealed considerable detachment forces (median detachment forces of all cycles ranging from 0.00 mN to 22.73 mN). Except for toluol, all solvents inhibited adhesion in all cycles of solvent treatments. Toluol, in contrast, also revealed high detachment forces in all subsequent measurements independently on the treatment. The detachment forces first increased to 560.35 ± 255.57 mN (3rd cycle) and then leveled out at a median detachment force of 272.15–308.05 mN independently on the treatment. In all cycles except the first, toluol treated eggs had large attachment ratios (75–92%). All solvents generally revealed high attachment ratios as well, except for the first cycle. Only very few cycles revealed attachment ratios below 50% (see Figure 6). The reversible inhibition by other solvents caused a strong reduction of the force. For many eggs, the measured adhesion was still sufficient to attach the eggs (*SF* higher than 1). However, the detachment forces of the solvent treated eggs were always much lower than those of the eggs treated with water. The solvents additionally had a strong effect on the adhesion of the water treated samples as well. 

Although all solvents except for toluol revealed a strong increase of detachment force in the water treated measurements, the detachment forces revealed differences for different treatments, either regarding the actual force per cycle, but also in the progression from cycle to cycle. Acetic acid caused the lowest median detachment forces for the water-based measurements and similar values from cycle to cycle (15.05 to 52.36 mN). The solvents had either increasing or decreasing effects on the detachment forces from cycle to cycle. The median detachment forces of the water treated eggs decreased for the solvents acetone (from 1195.48 mN to 67.82 mN) and hexane (from 751.74 mN to 63.08 mN). However, on the one hand, the detachment force of the acetone treated eggs was higher in the first water-based cycle in comparison to the hexane treated group, but the detachment force decreasing effect of acetone was stronger than that of hexane (Figure 6). Increasing median detachment forces of water treated eggs are revealed in the solvents chloroform (from 72.05 mN to 822.66 mN) and propan-2-ol (82.20 mN to 255.59 mN). Although both solvents revealed similar detachment forces during the first water cycle, the increase was stronger for chloroform (see Figure 6). The detachment forces during water cycles increased for propan-2-ol treated samples only to the maximum in the 3rd water cycle and then decreased again.

**Figure 7:**
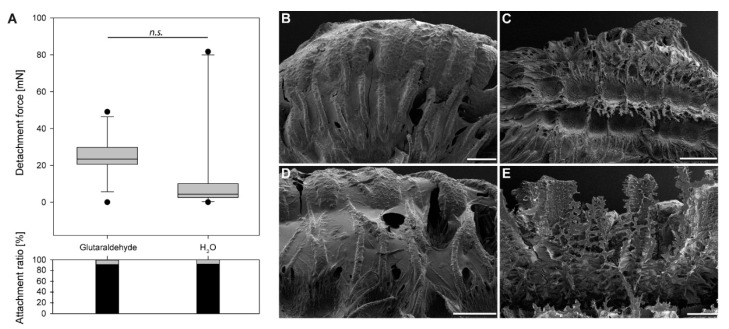
Detachment forces of glutaraldehyde treated eggs and subsequently water exposed eggs: (**A**) Boxplots of the detachment forces of both treatment cycles (above) and the corresponding attachment ratio (below). The horizontal line represents the median, the upper and lower borders of the boxes represent 25 and 75 percentiles and the whiskers represent 10 and 90 percentiles, *n.s.* = not significant (Wilcoxon Signed Rank Test, *N*_1,2_ = 12, *p* = 0.23); (**B**–**E**) Scanning electron micrographs of the adhesive system of *P. philippinicum* eggs exposed to (**B**) glutaraldehyde, (**C**,**E**) toluol, (**D**) hexane. Scale bars: 500 µm (**C**), 200 µm (**B**,**D**,**E**).

As glutaraldehyde is a strong cross-linking fixative, this chemical was used for treatment as well, but just for one cycle with the chemical and one subsequent cycle with water. Nearly all eggs attached after treatment with either glutaraldehyde or water (Figure 7A). The detachment forces for both treatments were rather low and measured 23.4 ± 11.9 mN for glutaraldehyde treated samples and 4.32 ± 29.24 mN for water. The difference was found to be not significant (Wilcoxon Signed Rank Test, *W* = 32, *T*+ = 55, *T*− = 23, *Z* = 1.25, *N*_1,2_ =12, *p* = 0.23).

#### 3.2.2. Comparison of Maximum Detachment Forces

**Figure 8:**
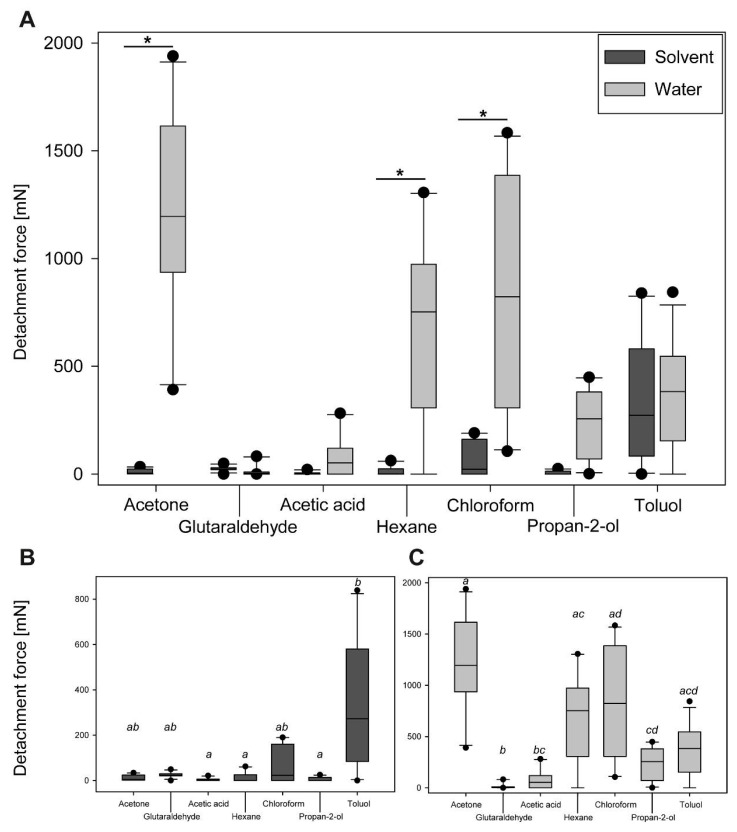
Maximum detachment forces of eggs for all solvents and their corresponding maximum detachment force of the water treatment during the cross-treatment experiments: (**A**) Boxplots for all solvents and the water treatment. The cycle with the highest median detachment force has been picked for each treatment. Statistical difference is shown only between the solvent and the corresponding water treatment (Kruskal–Wallis ANOVA on ranks, *p* ≤ 0.001). * = *p* < 0.05 (Dunn’s post hoc test). Differences between the solvents, or the water treatments, respectively, are shown in (**B**,**C**); (**B**) Comparison of maximum detachment forces for the solvents cycles of the sequential cross treatment experiments; (**C**) Comparison of maximum detachment forces for the water cycles of the sequential cross treatment experiments; The horizontal line represents the median, the upper and lower borders of the boxes represent 25 and 75 percentiles and the whiskers represent 10 and 90 percentiles. Groups with the same lowercase letter are statistically not different (Kruskal–Wallis ANOVA on ranks, Dunn’s post hoc test, *p* < 0.05).

The detachment forces of eggs were rather different for different solvents and water cross effects for both, the different treatments and the different cycles per treatment. The overall attachment performance was compared using the maximum detachment force among the different cycles per solvent (see Figure 8). The cycles with the highest median detachment forces were selected from the cross experiments for all solvents and their corresponding water treatments. Generally, the water treatment generated more adhesion than the solvent-based treatment (Figure 8A). However, statistical analysis revealed significant differences just for the water treatment of three solvent groups: acetone, chloroform and hexane (Kruskal–Wallis ANOVA on ranks, *H* = 86.59, d.f. = 13, *p* ≤ 0.001; Dunn’s post hoc test, *p* < 0.05). The other solvent groups generated the same adhesion for both the solvent and the water treatment (Dunn’s post hoc test, *p* > 0.05). The mediated detachment force was much larger for water-treated eggs in comparison to the solvent-treated eggs, if there was a significant difference (acetone: 1195.5 mN vs. 4.4 mN; hexane: 751.7 mN vs. 0.8 mN; chloroform: 822.7 mN vs. 22.7 mN). Considering the detachment forces in the solvent treated groups only, the detachment force was the highest for toluol (272.15 ± 272.72 mN), and statistically significantly higher than in most other groups (Kruskal–Wallis ANOVA on ranks, *H* = 26.86, d.f.= 6, *p* ≤ 0.001; Dunn’s post hoc test, *p* < 0.05). Particularly the detachment force generated by toluol was significantly higher than that of acetic acid (1.36 ± 6.14 mN), propan-2-ol (0.00 ± 8.59 mN) and hexane (0.81 ± 20.96 mN; Dunn’s post hoc test, *p* < 0.05). The detachment forces generated by treatment with acetone (4.37 ± 12.41 mN), glutaraldehyde (23.43 ± 11.40 mN) and chloroform (22.73 ± 80.30 mN) showed no statistical difference to any of the other groups (Dunn’s post hoc test, *p* > 0.05). The water-based treatments of the eggs produced different degrees of adhesion depending on the preceding solvent treatment (Figure 8B). The eggs pre-treated with acetone revealed the highest detachment forces during the cross tests with water (1195.48 ± 447.80 mN). These eggs adhered statistically significantly better than those in most other pre-treatments (Kruskal–Wallis ANOVA on ranks, *H* = 42.78, d.f. = 6, *p* ≤ 0.001; Dunn’s post hoc test, *p* < 0.05), with the exception of hexane (751.74 ± 425.23 mN), chloroform (822.66 ± 501.19 mN), and toluol (381.89 ± 248.85 mN; Dunn’s post hoc test, *p >* 0.05). The lowest detachment forces were measured for eggs pretreated with glutaraldehyde (4.32 ± 27.99 mN), which were also significantly lower than for all other solvents (Dunn’s post hoc test, *p* < 0.05), except for acetic acid (52.36 ± 91.83 mN; Dunn’s post hoc test, *p >* 0.05). 

## 4. Discussion

The systematic exploration of the influence of solvents with different chemical properties on insect glues with regard to their adhesive performance is rarely carried out. To our knowledge, none of the experimentally tested insect glues have been examined in regard to their interactions with different solvents. Usually, insect glues are not water-soluble and presumably chemically stable [48,60]. Some exceptions are the eggs of dragonflies and mayflies [48,49,50,51,52,53,54,55,56,57,58,59]. These, however, are normally deposited into the water, the habitat of the larvae [50,80]. The adhesive system of *Phyllium philippinicum* eggs, however, is different. The females deposit eggs terrestrially. They are flicked away and once activated by contact with water develop adhesion [22]. In the following, we will discuss the responses of this adhesive system to the exposure to different chemical influences.

### 4.1. Morphological Responses 

Water exposure triggers two main responses of this adhesive mechanism: pinnae expansion and glue liquefaction [22]. The pinnae fan out after contact to water and adapt to the substrate surface profile, while the liquid glue is transported onto the substrate and spread out by the expansion of the pinnae. Different solvents had different effects on both the expansion of pinnae and the behavior of glue (Table 2). In our previous study, only water was used to trigger the expansion of the pinnae [22]. In the present paper, this effect was likewise achieved in most water-based solutions and polar solvents. All nonpolar solvents did not initiate pinna expansion. Seemingly, the expansion of the pinnae is dependent on polar molecules, like water. Generally, during tanning of insect cuticle water is removed from the proteins and cross-links are formed (see, e.g., in [81,82]). These cross-links increase the stiffness of the cuticle as they decrease the swelling capability of proteins [82]. Similar mechanism could be involved in the expansion of the pinnae as well. Polar molecules potentially mask the polar components in the proteins and hydrate the material of the pinnae. This could explain why aprotic solvents had no effect on the expansion the pinnae and inhibited the process of inflation of the appendages. Furthermore, propan-2-ol did not trigger the expansion of pinnae, although ethanol generated at least a reduced expansion (Figure 3). Because of the larger nonpolar carbohydrate part of propan-2-ol (C_3_H_7_OH) in comparison to ethanol (C_2_H_5_OH), propan-2-ol is less polar than ethanol [83]. Therefore, the effect of the polar part might be even more reduced. Glutaraldehyde is a common fixative for biological samples including insect cuticle, and it introduces intermolecular cross-links in proteins as well [84,85], similar to the nonpolar solvents, which can also explain the absence of expansion of the pinnae. Most polar solutions initiated an expansion of the pinnae except for the solution with a pH of 10.9. However, the pinnae under influence of pH 13.9 solution did expand. 

In detail, the response of the glue did not follow the same pattern. The strong solvents acetone and chloroform were able to solve the glue from the surface of the egg (Figure 5C,F). This agrees with the assumption of Büscher et al. 2020 [22], that the glue possesses (at least) two functional units, of which one is nonpolar and attached to the surface of the egg. The other is polar and faces exteriorly. Polar solvents exhibit large dipole moments, because of the difference in electronegativity of the contained atoms. Polar protic solvents often contain an -OH group, and thus can form powerful hydrogen bonds. Such solvents, for example, ethanol, propan-2-ol, and H_2_O, can dissolve polar solutes [86]. Increasing carbohydrate chains presumably play a role here as well; therefore, it is likely that the same effects cause the gradual decrease of liquefaction among the three solvents, with propan-2-ol not being able to solve the polar component of the glue. Non-polar solvents, on the other hand, possess low dielectric constant values as electric charges in these molecules do not tend to be evenly distributed. These solvents are hydrophobic and lipophilic, like the ones reported before, and can detach the glue from the egg [86]. 

Most other permanent adhesives on the eggs of insects are reported to be proteinaceous [60,64,65,66,67,68,69,70,71,72,73,74], and previous research suggested a glycoprotein as a potential candidate for the glue presented here [22]. The described effect of two functional subunits would be explained by this [60,63,69,71,72,73]. Judging on the SEM analysis, pH values except for 7.0 had a strong effect on the morphology of the adhesive and caused the glue to denature. The pH of 7.0, however, had the same effect (and the same pH value) as the water used in [22], with proper expansion of the pinnae and regular liquefaction of the glue. Glutaraldehyde, hexane and propan-2-ol had no effect on the glue morphology. 

The choice of the solvent had a strong effect on the behavior of pinnae and glue. This also implies a distinct effect on the attachment performance. The spreading of the glue (degree of liquefaction), the area covered by the adhesive system (pinnae expansion), as well as the deactivation of the adhesive played a role for attachment generation. The fundamental effects of these factors are visible in the influence of the solvent choice on the attachment over repetitive detachment cycles. The eggs of other phylliid taxa bear similar exochorionic structures as well, see, e.g., in [4,15,16,17,18,22]. These species potentially exhibit adhesive mechanisms as well, as the appendages on the eggs respond to water contact, like in *P. philippinicum*. Whether these indeed reveal a glue as well, and particularly whether this glue has a similar composition, is so far unreported. Future studies could investigate in the morphological comparison of the different pinna morphology of different species, the presence of an adhesive fluid and can potentially elucidate the effects of the different structures for species-specific adaptations towards specific substrates. This could furthermore guide to experimental studies on the attachment performance on different natural substrates. Such experiments can also include, next to the bark or leaf surfaces of the actual foodplants, attachment measurements on fur of mammals, which could potentially serve as carriers of the eggs and for dispersion via zoochory, as observed in plant seeds [23,24,25,26,38,39,40] and the eggs of other insects [46,47,48].

### 4.2. Adhesion Performance during Sequential Testing 

The measured detachment forces were influenced by the solvent effects on the pinnae and the glue described above. Solvents which strongly inhibited the performance of the adhesive system and, hence, resulted in no adhesion during the initial cycle, are discussed below (see Section 4.3). The progression of the detachment forces during repetitive measurement cycles of the solvent with pH 7.0 was similar to that reported in [22]. Distilled water (see in [22]), as well as the pH 7.0 solution, caused an increase of the detachment force during the first repetitive measurements and a subsequent fading out of the adhesion. The glue responded to different pH regimes, while a pH of 7.0 revealed the same results as pure distilled water: the function of the glue was largely affected in all other pH regimes. This can partly be explained by the reaction of the glue. While the neutral solution like water liquefied the glue, the glue aggregated in all other pH regimes. This partly supports the assumption that the glue is protein-based [22], as many proteins have a pH optimum around 7.0 [87]. However, structural aggregation of the glue, as observed in the SEM, does not completely correlate with the functionality. While low pH values indeed revealed low initial adhesion and low detachment forces in the subsequent measurement cycles (Table 2), a pH of 13.9 enabled even stronger initial adhesion followed by a sudden decrease of the detachment forces in the repetitions (Figure 2B). Although the structural stability of proteins is affected by the pH, the function might follow different trends [87]. To achieve proper adhesion, the liquid glue would make contact with the adjoining substrate and spreads into surface asperities to create sufficient contact area for adhesion [75,88]. The aggregation in the different pH regimes other than pH 7.0, therefore, potentially hinders adhesion, as proper spreading necessary for strong adhesion [42,43] is inhibited. The sudden drop in detachment force in the sequential detachment events of eggs, treated with pH 13.9 and pH 5.0, might then be a result of the aggregated protein that still generates adhesion to the substrate, but less adapts to the substrate profile. Therefore, it can only make a proper contact in the initial contact formation, but retain the initial form for the following measurements. Furthermore, the function of the protein is dependent on the sequence and amount of different amino acids [89], which might differently respond to different pH [87]. This could enable two different optima for the same protein, depending on the actual composition, which would also imply a complex structure of the protein content of the glue. However, the insights gained for the structure of the glue remain ambiguous, and strongly suggest an examination of the molecular structure. Furthermore, the glue might contain non-proteinous components as well, that do respond to different pH values than the protein. 

With water as a solvent, the pinnae extend after contact and enforced adaption to the texture of the corresponding surface [75,90]. This effect is visible in ethanol and acetone as well, but to a different extent (Figure 3), which influences the glue performance over several cycles of detachment events as well. Furthermore, these two solvents have different effects on the glue. Using only acetone as a solvent, without cross treatments, the pinnae expand, but most of the glue remains on the egg surface. However, the detachment force is low for all cycles, though sufficient (*SF* > 1) and increases over subsequent cycles. The detachment force of ethanol-treated eggs increased over the cycles, as well, but to a stronger extent. As the pinnae expand less under the influence of ethanol, in comparison to acetone (Figure 3), the stronger adhesion is probably a result of the glue behavior. In both solvents, a dissolution of the solvent from the egg surface, but retaining on the pinnae, can probably continuously accumulate glue in the interface of egg and substrate, and thus increase the attachment over the cycles, by generating a larger contact area [75,88]. 

Another aspect potentially interfering with the generation of large contact area could be a product of the high volatility of some of the solvents. The glue of these eggs hardens due to evaporation of the solvent [22]. As both water and ethanol are quite volatile [91], acetone more than ethanol, and evaporate rapidly from the eggs, it might be the case that the solvent was not able to play an essential role in enabling spreading of the glue. The adhesive was partially dissolved in this case and the solvents that evaporate quickly highly reduce the exposure time of the glue to the solvent, and in turn reduce the potential of the glue to spread over the substrate. In this case, reduced contact area between the glue and substrate consequently leads to an adhesion reduction. However, the detachment force increased later on, probably, because the repeated solvent treatment increased the effective duration of exposure and the glue accumulated on the tips of the pinnae with the repetitive treatments.

### 4.3. Adhesion Performance during Cross-Testing 

Interestingly, none of the used chemicals irreversibly deactivated the glue. After treatments by any solvent in experiments with alternating solvent/water treatments, the strongly increased adhesion was always measured after the subsequent water treatment. Considering the measurements after the solvent treatments only, toluol stands out of most other solvents, as the adhesion was much stronger than without water treatment in between. Furthermore, this is the only solvent that resulted in a strong adhesion of the egg itself. The glue was detached from the egg, probably by dissolving the nonpolar portion of the glue by the nonpolar toluol [72]. However, the glue was not completely washed off from the egg and accumulated on the pinnae (Figure 8C,E). This resulted in its better positioning of the glue. Subsequent treatment with water activated the glue. In contrast to the other solvents, the treatment with toluol generated adhesion even stronger than after the water treatment in the meantime. Potentially, the water might have prepositioned the glue and subsequential toluol treatment has activated the nonpolar compounds of the glue which then bridged between the egg and the substrate.

During all other cross-treatments, the solvents mostly deactivated the adhesive. However, the detachment forces of the water-based cycles revealed three different main effects depending on the solvent: (1) increasing mean detachment forces over the cycles with water treatment, (2) decreasing mean detachment forces over the cycles, and (3) almost constant detachment forces (Figure 6). The peak detachment forces of different solvents, even with the same main effect, differed among the solvents. Acetic acid deactivated the adhesive system as described above. However, the detachment forces with water were constantly low, probably because the deactivated glue remained largely denatured and was therefore partially functionalized. Still, in the water-based treatments, the adhesion was sufficient to hold the eggs on the substrate (mean *SF* ≈ 360). All other solvents revealed stronger effects on the water-treatment cycles, though to different extents. Increasing detachment forces over the cycles are result of accumulation of the glue on the egg–substrate interface as it was observed for propan-2-ol and chloroform. However, the reduced overall detachment force, measured for propan-2-ol pretreated eggs (in comparison to water as the only solvent of the glue), can be result of the higher volatility of propan-2-ol [91] and its reduced polarity [83]. Chloroform, in contrast, strongly increased the adhesion over repeated cycles of attachment, detachment, and subsequent reattachment due to the accumulation of the glue. Chloroform could dissolve the glue from the egg (Figure 5C), because of the non-polarity of the solvent, but not completely washed the glue off, because of the high volatility of the solvent [91] and the surface enhancing effect of the pinnae. With water contact, the pre-spread glue is liquefied and hence generates a large contact area, resulting in a stronger adhesion [88,92]. This effect progressively amplified over the treatment cycles. Decreasing detachment forces, however, are result of the glue pre-spreading as well. Acetone, in particular, revealed increased adhesion in the first water cycle of the cross experiments (Figure 6). According to the SEM analysis, large amounts of the glue were washed off from the egg surface (Figure 5F). The glue was seemingly kept by the pinnae and remained on the egg surface. Acetone on its own was not able to activate the glue properly, but it did not damage its molecular structure, because the subsequent water treatment liquefied the glue, which was detached from the eggshell. This caused a high amount of glue mobilized at once, making contact with the substrate. After the first cycle of high detachment forces, adhesion dropped strongly in the next water-based cycle, because the majority of the glue remained on the substrate and therefore was not available for further attachment cycles. This would normally be avoided by the attachment of the glue to the eggshell [22], which was overcome by the effect of the acetone. As the glue is most likely not produced by the egg [22], the decrease in adhesion is a consequence of the loss of glue that remained on the substrate. Pure hexane treatment did not have an effect on the morphology of the glue (Figure 7D), however, revealed similar effects on the detachment forces as acetone. However, hexane is even more volatile [91] and probably was not able to reach much the nonpolar parts of the glue and, as a nonpolar solvent, did not activate the polar groups on the molecule [91]. Therefore, the decrease of the detachment forces here was reduced in comparison to acetone. 

As explained above (see Section 4.1.), glutaraldehyde is a cross-linking fixative and surprisingly did reveal rather strong adhesion in both treatments (Figure 7A), although a cross-linking effect would have been expected on the glue as well [84,85]. However, the pinnae did not expand and the glue was not largely affected besides minor spreading along the pinnae (Figure 7B). As the safety factor was considerably high for glutaraldehyde, during the repetition with water as a solvent, apparently the minor amount of active glue was sufficient for generating adhesion (median *SF* ≈ 170) on the one hand, but most of the glue was not mobilized properly, on the other hand. The median *SF* for the detachment force of the solvent cycles with the strongest adhesion revealed the highest value for toluol, with an *SF* of ~4100. This is almost as strong adhesion as reported for water treated eggs on hydrophilic substrates (4825 [22]). The effects described above resulted in increased *SF*s in water-based cycles with pretreatment by the solvents acetone (*SF* ≈ 8800), chloroform (*SF* ≈ 6000), and hexane (*SF* ≈ 5500).

Effect of solvents and pH on the stability of the egg’s chorion were not investigated herein. At least some layers of the chorion are mineralized [93,94] and would be particularly vulnerable to low pH solutions. However, if the pinnae sustain a loss of stability, they are more easily ripped off along with the glue. The investigation of factors influencing the mechanical integrity of the eggshell and consequently the effect on the adhesion as a consequence of pinnae ripping off would be interesting for further studies. 

The egg glue of *P. philippinicum* endures various chemical treatments and retains its adhesive function after subsequent water contact. The solvents explored herein provide much harsher influence on this adhesive system than the expected influences of the natural conditions for these eggs. Presumably, surfaces available for attachment of these eggs are covered with fluids with less extreme pH and less strong solvents. Consequently, the surface topography and chemistry are essential to adapt to for insects, as shown for the same species [22] and other insects [22,41,42,43,44,60,61,62]. However, the putative complex composition of the glue of *P. philippinicum* enables them to withstand a broad range of chemical influences and retain its adhesive capability, as water is prevalent in the tropical rainforests inhabited by this species [95]. Various other egg attachment systems evolved in Phasmatodea as well [2,3,96]. These originated from close coevolution between plants and phasmids during the radiation of this insect lineage [21] and were optimized to master different attachment problems. As similar mechanisms evolved convergently in different groups of phasmids [2,21,31], these might target similar problems with different solutions. Analyzing the constraints underlying the functional aspects of problem-specific solutions in biological systems can help to find proper solutions for further technical applications [75].

### 4.4. Biomimetic Considerations 

Insect egg glues are generally promising candidates for bioinspiration, particularly in the field of biomedical applications, because of their biodegradability and possible biocompatibility [60]. Often the exploration of bioadhesives is carried out to target three main factors for the improvement of industrial glue systems: contact reliability, environment friendliness, and reduction of the necessary amount of glue [75,97]. 

Usually, fiber reinforcement is used to increase the mechanical stability of adhesive systems [98,99]. The fibers are embedded into the glue and provide a framework which is stronger than glue itself. While the glue often is not that mechanically stable, failure of an adhesive system is often a result of the material failure of the glue (cohesive failure) [100]. The fibers reinforcing the adhesive reduce the risk of material failure of the overall system and increase the mechanical stability of it. In the system, exemplified by the eggs of *P. philippinicum*, the fibers which are mantled by the glue serve further purposes. On the one hand, the hierarchical structure of the pinnae facilitates an adaption to the surface roughness of the substrates [75,89], and, furthermore, serves a proper spreading of the glue. Glue brush applicators (e.g., US patent USD776939S1) exploit a similar mechanism for dispersion of glue and filling of challenging surface asperities [101]. The pinnae also increase the versatility of the adhesive system and contribute to the compensation of chemical influences on the adhesive system, on the other hand. Furthermore, the glue itself can synergistically interact with the pinnae. As hypothesized for the glue of the species examined herein, a combination of two different functional chemical groups on the two ends of the molecule can provide a reliable bonding with the substrate, by attaching the molecule to the substrate with the one hand, and strong bonding to the egg surface on the other hand. This combination also enables the functionality of the adhesive system, even in chemically challenging environments, like in presence of solvents, by keeping residuals of the glue on the surface of the pinnae. The hierarchical structure of the pinnae can also increase the overall surface area of the egg and, therefore, increases the probability of glue residuals to be trapped on the surface of the egg. Additionally, the combination of fibers and a multi-functional glue that can be reactivated by an external stimulus, can provide potential for self-adjustment. The reversibility of the glue, on the one hand, provides the possibility to optimize the deposition of the egg in the proper environment for incubation [22], but on the other hand, the pinnae increase the prolonged availability of the glue on the egg surface. 

We believe that such a combination of the multi-functional glue and fiber reinforcement with specific surface chemistry, that interacts with the glue, can potentially inspire biomimetic approaches that target the use of versatile glues for multiple purposes, long term applications and self-adjusting responsive glue systems [100,101,102,103]. It might potentially even inspire technological applications leading to an optimization of the glue amount used to a necessary but satisfying minimum, resulting in reduced material cost and therefore to developments of more sustainable adhesive systems [60,100,104,105]. 

## 5. Conclusions

The adhesive system of *P. philippinicum* consists of two main components: the pinnae and the glue. Both components synergistically provide an elaborate mechanism to deal with numerous environmental influences and achieve self-optimization of adhesion produced by this system. The fibrous pinnae do not only reinforce the action of the glue, provide additional mechanical stability to the bond, and facilitate adaption to the substrate texture, but also enable keeping the glue on the egg, even in the presence of strong solvents. The glue itself generates versatile adhesion in different chemical regimes and facilitates the reuse of detached glue on the egg surface. The combination of at least two functional domains provides adhesion to both hydrophilic and hydrophobic substrates and the surface of the egg itself. In comparison to the detachment forces obtained after water treatment, high pH generated similar high detachment forces. Strong solvents were able to detach the glue from the egg surface, but as the pinnae kept the glue on their surface, subsequent treatment with water even increased adhesion. Although some solvents inhibited adhesion in the first place, the inhibition was reversible in all tested cases and the glue retains its functionality in an astonishing range of chemical influences with the sufficient effect in nearly all cases. The combination of such a multifunctional adhesive and the supporting structures of the egg surface can potentially inspire biomimetic approaches aiming at optimizing adhesive systems, particularly in face of unpredictable chemical environments, or/and at enabling reduction of the glue amount. The glue itself might also be a potential candidate for mimicking it as a biodegradable adhesive for a biomedical use.

## Figures and Tables

**Table 1:** List of the used solutions, including specifications of eggs and experimental cycles.

Solvent	Solvent Group	Cycles	Sequential (*n*_eggs_, Weight ^1^)	Cross (*n*_eggs_, Weight ^1^)
Acetic acid (pH 2.9)	pH (protic)	10/8 ^2^	10, 13.45 ± 1.03	10, 13.00 ± 1.77
pH 5.0 ^3^	pH (protic)	10	10, 13.5 ± 1.0	no
pH 7.0 ^3^	pH (protic)	10	20, 14.45 ± 1.14	no
pH 10.9 ^3^	pH	10	10, 14.25 ± 1.12	no
KOH (pH 13.9)	pH	10	10, 13.70 ± 0.79	no
Acetone	polar-aprotic	10/6 ^2^	10, 13.95 ± 0.89	10, 14.35 ± 0.93
Chloroform	nonpolar-aprotic	10/6 ^2^	no	10, 14.70 ± 0.92
Ethanol	polar-protic	10	10, 14.30 ± 1.14	no
Glutaraldehyde (25%) ^4^	fixiative	2	no	12, 13.85 ± 0.95
Hexane	nonpolar-aprotic	6	no	10, 13.45 ± 0.89
Propan-2-ol	polar-protic	8	no	10, 13.45 ± 1.18
Toluol	nonpolar-aprotic	6	no	12, 15.30 ± 1.88

^1^ median ± s.d. in mg. ^2^ Sequential/cross. ^3^ Mixture of acetic acid and KOH. ^4^ all other solvents were not diluted.

**Table 2:** Responses of the egg adhesive system to the exposure to different solvents. Pinnae expansion as shown in Figure 3. Glue behavior: denaturated, aggregation of the glue; liquefied, glue mobilization as described in [22]; solved, glue detached from the egg surface; Initial detachment force: s.d., standard deviation; - means 0.0 mN detachment force; Effect of replication: change of the detachment forces over the next cycles; Effect of H_2_O-treatment: activation, higher attachment forces after no attachment with solvent treatment; initiation, higher attachment forces after first water treatment, but with subsequent solvent treatment still adhesion; n.a., not measured.

Solvent	Pinnae Expansion Behavior ^1^	Glue Behaviour ^2^	Initial Detachment Force (mN, Median ± s.d.)	Effect of Replication	Effect of H_2_O Cross Treatment
Acetic acid (pH 2.9)	expansion	denaturated	-	no change	activation
pH 5.0	expansion	denaturated	3.18 ± 9.39	de-, then increase	n.a.
pH 7.0	expansion	liquefied	310.48 ± 278.33	in-, then decrease	n.a.
pH 10.9	inhibited ^3^	denaturated	0.477 ± 3.67	no change	n.a.
KOH (pH 13.9)	expansion	denaturated	479.78 ± 310.43	decrease	n.a.
Acetone	expansion	solved	1.80 ± 0.69	de-, then increase	activation
Chloroform	inhibited ^3^	solved	-	no adhesion	activation
Ethanol	moderate expansion	liquefied	1.80 ± 52.74	increase	n.a.
Glutaraldehyde	inhibited ^3^	no change	23.43 ± 11.40	n.a.	activation
Hexane	inhibited ^3^	no change	-	no adhesion	activation
Propan-2-ol	inhibited ^3^	no change	-	no adhesion	activation
Toluol	inhibited ^3^	solved	-	no adhesion	initiation

^1^ Figure 3B–D. ^2^ Figure 5. ^3^ reversible with water contact.
